# Hypothesis: Paralog Formation from Progenitor Proteins and Paralog Mutagenesis Spur the Rapid Evolution of Telomere Binding Proteins

**DOI:** 10.3389/fgene.2016.00010

**Published:** 2016-02-10

**Authors:** Arthur J. Lustig

**Affiliations:** Department of Biochemistry and Molecular Biology, Tulane University, New OrleansLA, USA

**Keywords:** telomeres, evolution, non-LTR reverse transcription, telomerase, models, stress response

## Abstract

Through elegant studies in fungal cells and complex organisms, we propose a unifying paradigm for the rapid evolution of telomere binding proteins (TBPs) that associate with either (or both) telomeric DNA and telomeric proteins. TBPs protect and regulate telomere structure and function. Four critical factors are involved. First, TBPs that commonly bind to telomeric DNA include the c-Myb binding proteins, OB-fold single-stranded binding proteins, and G-G base paired Hoogsteen structure (G4) binding proteins. Each contributes independently or, in some cases, cooperatively, to provide a minimum level of telomere function. As a result of these minimal requirements and the great abundance of homologs of these motifs in the proteome, DNA telomere-binding activity may be generated more easily than expected. Second, telomere dysfunction gives rise to genome instability, through the elevation of recombination rates, genome ploidy, and the frequency of gene mutations. The formation of paralogs that diverge from their progenitor proteins ultimately can form a high frequency of altered TBPs with altered functions. Third, TBPs that assemble into complexes (e.g., mammalian shelterin) derive benefits from the novel emergent functions. Fourth, a limiting factor in the evolution of TBP complexes is the formation of mutually compatible interaction surfaces amongst the TBPs. These factors may have different degrees of importance in the evolution of different phyla, illustrated by the apparently simpler telomeres in complex plants. Selective pressures that can utilize the mechanisms of paralog formation and mutagenesis to drive TBP evolution along routes dependent on the requisite physiologic changes.

## Introduction

Telomeres, the DNA-RNP structures present at the termini of all eukaryotic chromosomes, are essential for genome stability and function. The telomere serves two functions that are fundamental for viability. The first is to provide a solution to the end-replication problem. This problem refers to the inability of the lagging strand DNA of semi-conservative replication to maintain its terminal RNA primer at the 5′ end of any replicating linear molecule (Levy et al., 1992). The leading stand, in contrast, creates a blunt ended telomere at the other terminus. The lagging strand will form one 3′ overhang terminus (Kazda et al., 2012; Bonetti et al., 2013; Ghodke and Muniyappa, 2013). Continuing rounds of semi-conservative replication will result in the loss of DNA primers, leading to attrition and chromosome loss. The processing of the blunt-ended telomere is variable in different organisms (Kazda et al., 2012; Bonetti et al., 2013). Regardless, the loss of a terminal DNA primer predicts the inevitable attrition of terminal sequence, and, ultimately, cellular inviability.

The solution to this problem is based on the terminal 3′ overhang that serves as a substrate for recombination or telomerase. Telomerase is the RNP-reverse transcriptase that adds G + T-rich simple sequence onto the 3′ terminus using the RNA as a template. The core enzyme rate and processivity are regulated by a multiplicity of holoenzyme components and telomere binding proteins (TBPs; Tucey and Lundblad, 2014; Vogan and Collins, 2015). Telomerase can catalyze addition in a processive or a distributive mechanism. The repeats added are most often identical, but, in some organisms (e.g., fungal systems) can add inexact repeats. The irregular repeat is thought to be formed by misalignment of DNA on the RNA template (Petrov et al., 1998; Forstemann and Lingner, 2001). As an example of holoenzyme regulation, the budding yeast Cdc13 protein associates with and recruits the auxiliary protein, Est1. Est1, in turn, recruits the telomere reverse transcriptase (TERT, Est2 in yeast) and the complex with the RNA subunit (TR) finally recruits the Est3 subunit (Tucey and Lundblad, 2014).

The second function of a telomere is to overcome the end-protection problem (de Lange, 2009). That is, the telomere must not be accessible to non-specific enzymes, including nucleases, ligases, and recombinases that may lose, destabilize, or rearrange the telomere, respectively. In this sense, the telomere is a cap against activities that lead to genomic dysfunction, while allowing the access of positive and negative regulators of telomere addition.

One protective function is the feedback regulation of telomere size that is present in all organisms, although the mechanisms may vary (Evans and Lundblad, 2000). In yeast, a competition between negative and positive regulators of telomerase form a steady state using the ATM pathway (Lustig and Petes, 1986; Bianchi and Shore, 2007; Sabourin et al., 2007; Hirano et al., 2009; Martina et al., 2012; Ribeyre and Shore, 2012). ATM (Tel1) normally arrests cells, in response to double strand break, in the G2 phase of the cell cycle, until repair of DSBs is complete (Usui et al., 2001). However, the telomeric DSB is protected from both repair and genomic instability in part by this equilibrium creating an “anti-checkpoint,” a part of the telomeric cap function (Carneiro et al., 2010).

In duplex DNA, the telomeric protein Rap1 forms the basic telomeric chromatin in yeast (Wright et al., 1992). Some of the major TBPs (e.g., in yeast Rap1 and the yKu70/80 heterodimer) protect the telomere from non-homologous end joining and inhibit end fusion (Frank-Vaillant and Marcand, 2002; Pardo and Marcand, 2005). Another cap structure, the Cst1/Stn1/Ten1 (CST) complex, also serves as a physical cap. Telomerase also appears to have the ability to block the end of the telomere (Blackburn et al., 2000). Finally, in ciliates, Hoogsteen base-paired G4 structures, such as the G-quartet, associate with TBPs and telomerase to both act as a cap and as a regulator of telomerase addition *in vitro* and *in vivo* (Fang and Cech, 1993a; Oganesian et al., 2006). Taken together, the activities of homeostatic factors, telomerase, capping proteins, and G4 DNA TBPs control telomere size in context of the cell cycle.

The ATR pathway, however, is another part of the telomeric DNA checkpoint control. If telomerase does not add a compensatory amount of G + T repeats, cells will begin to senesce (Abdallah et al., 2009). If the telomere shortens beyond a threshold size, the cells will undergo a G2 arrest and a further loss of telomere sequences mediated by both recombinational and replicative DNA damage, leading to inviability. Ultimately, survivors use either a break-induced recombination or a rapid telomere elongation process to form elongated telomeres (Lustig, 2003; Pickett et al., 2011; Pickett and Reddel, 2012). The mechanistic details may differ along the evolutionary spectrum of organisms, but the basic paradigm remains unchanged. In this theoretical perspective, we will focus on the TBPs that associate with telomerase generated telomeres.

## The Diversity of Telomere Binding Proteins

Evolutionary biologists and telomere researchers have long tried to explain the wide diversity of many proteins involved in telomere function and structure (Linger and Price, 2009). Models for the evolution of different modes of telomere maintenance are beginning to show promise. The major modes of telomere addition are telomerase and non-LTR reverse transcriptases. Telomerase may have formed from non-LTR reverse transcriptases with a specificity high in G + T content (Garavis et al., 2013). In contrast, reverse transcriptase possibly continued to be used when target site sequence bias is absent. These may well be the primary ancestral mechanisms of telomere formation, although the ancestral origin is, by definition, a matter of speculation. Evolution may at times repeat previously used mechanisms. For example, *Drosophila* arose long after primordial telomeres, yet uses telomeric non-LTR retrotransposons that are (telomere specific, Villasante et al., 2008). The mechanism used in *Drosophila* may lend insights in an evolutionary context, with some caution that *Drosophila* may use a variation on a theme.

Most non-LTR retrotransposons appear to have formed degenerate heterochromatin that was subsequently maintained by recombinational mechanisms (Villasante et al., 2007). Recombinational activity is used in extant organisms as an alternative telomere pathway in the absence of telomerase (Louis and Haber, 1990; Preiszner et al., 1994; Mizuno et al., 2008; Li et al., 2009; Torres et al., 2011). Investigators have observed rolling circle replication, unequal sister chromatid exchange, and mechanisms of simple sequence elongation (Tomaska et al., 2004a, 2009; Torres et al., 2011). We cannot exclude these uncharacterized mechanisms in ancestral telomere formation.

The mechanisms of telomere elongation are presented to provide context. Our focus, however, will be on the exploration of the curious rapid evolution of the TBPs in the telomerase-based systems. These data are not consistent with either a simple movement toward complexity or simplicity during evolution (Gould, 1996; de Lange, 2015). The complexity of the plant genome and its sophistication in development do not explain the simplicity of its telomere with little difference between complex plants and algae. We feel that rapid TBP evolution can be explained by a set of basic principles that governs diversity.

### A Model for the Conservation and Diversity of TBF

#### Orthologs and Parologs

The major molecular biological means of describing closely related protein sequences is homology. However, the evolutionary significance of homology can be misinterpreted without a comparison among organisms of differing complexity. The significance of partial homology is difficult to interpret when applied to evolution. A protein having partial homology throughout all kingdoms and phyla tells us little about the directionality of inheritance during evolution. Homology and partial homology are anathema to many evolutionary biologists, providing information only about sequence identity, rather than evolutionary patterns.

The initial insights into evolutionary patterns were remarkable, having arisen independently of any knowledge of DNA. These theoretical and mathematical principles were based on abstract evolutionary concepts. The strongest hypotheses have weathered time to the genomic era. The field is finally in a position to test specific questions regarding the blueprints for telomere evolution at a molecular level.

Some specific terms that were last seen by most of us in a textbook require review. Two types of evolutionary relationships, orthologs and paralogs, are central to the outline of much of evolutionary change. The inheritance patterns and relative homology of proteins argue for a vertical process (as in an evolutionary tree) in evolution. In this way, a single ancestral progenitor can be envisioned by the orthologs among different organisms (Koonin, 2005).

**FIGURE 1:**
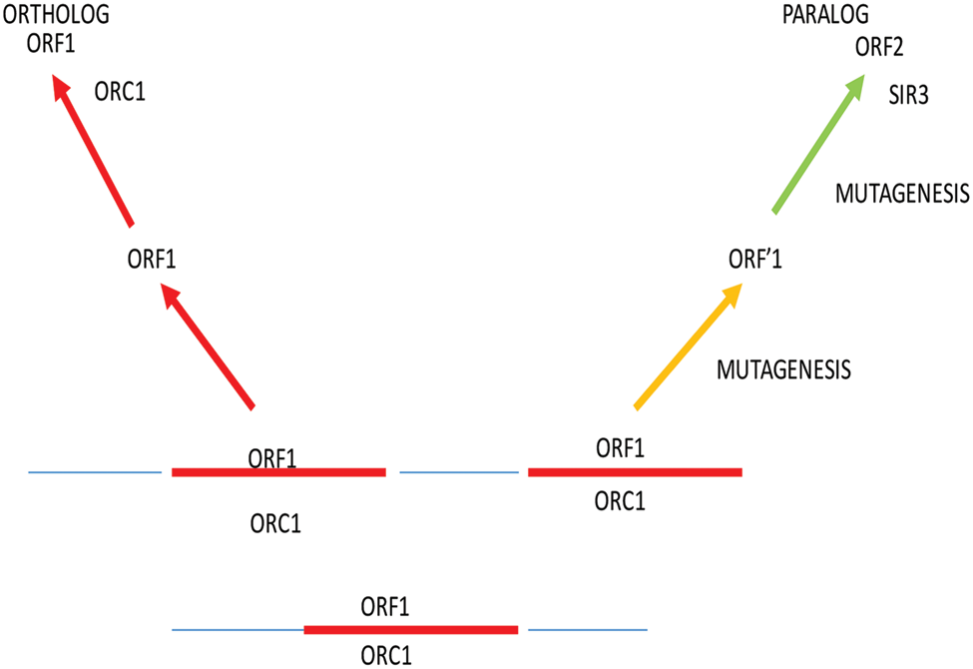
**Paralog formation and mutagenesis of a single ORF1.** Under conditions of stress response and high selectivity, recombination and mutagenesis increase the frequency of paralog formation during evolution. In this process, recombination results in duplication of the ORF1 coding sequence. The first copy, when separated by recombination, remains stable as ORF1. Under selection, the paralog undergoes an elevated level of mutagenesis caused by stress in response to dysfunctional telomeres. Unknown multiple rounds of mutagenesis take place in evolutionary time to ultimately give rise to a unique functional protein, ORF2. In the ORC1 example, paralog formation gives rise to Sir3, a protein involved in silencing of genes and the structure of telomeres in several yeast strains.

Paralogs, on the other hand, are protein products of DNA or genomic duplication that lead to horizontal evolution; particularly two duplicate proteins, one of which evolves from the progenitor in a unique direction under strong selective conditions (**Figure 1**). Sometimes, both paralogs evolve into new products. Ultimately, sequence and evolutionary analysis are required to provide more evidence for the existence of a paralog. This paralog can subsequently become an ortholog of a long line of species. Examples of telomeric paralogs are shown in **Table 1**. We propose that telomere dysfunction creates a variety of stress responses and selection pressures that use elevated paralog formation and mutagenesis that lead to an exceedingly high rate of TBP evolution.

**Table 1:** Examples of likely Tbp paralogs.

	Paralog1	Paralog 2	Paralog 3	Paralog 4	
*S. cerevisiae*	ORC1	SIR3			Liaw and Lustig, 2006
	EST1	EBS1			Zhou et al., 2000; Luke et al., 2007
	SIR2	HST1	HST2	HST3	SGD
	STN1^∗^	RPA2			Sun et al., 2009
	TEN1^∗^	RPA3^∗^			Sun et al., 2009
*Arabidopsis*	TRFL1	TRFL2	TRFL4	TRF4	Fulcher and Riha, 2015
		TRB1	TRP1	TRF9	
	PARP1	PARP2	PARP3		Boltz et al., 2014
	POT1a	POT1b			Cifuentes-Rojas et al., 2010
Rodentia	POT1a	POT1b			Hockemeyer et al., 2006
	PARP1	PARP2			Cook et al., 2002
	TRF1^∗^	TRF2^∗^			
Humans	EST1a	EST1b	EST1c		
	TRF1^∗^	TRF2^∗^			Broccoli et al., 1997
	STN1^∗^	RPA2^∗^			Bryan et al., 2013
	TEN1^∗^	RPA3^∗^			Bryan et al., 2013

*^∗^ Paralog was formed in an ancestor and subsequently maintained. Others appear to have formed in a particular species. Rodentia refers to mice and rat species. This is not a complete list and database homologies indicate that there are likely to be more TBF paralogs.*

### The Conserved Elements of TBP

There is great diversity among proteins that bind to telomeric DNA and that associate with other telomeric proteins or G4 structures. However, there is a subclass of proteins and DNA structures that are present in most organisms and serve a conserved function. Since these are important in any analysis, we will first discuss the highly conserved telomere capping proteins and DNA structures.

#### The Conserved MR (X/N) Complex

The primary roles of MRX in the signaling and processing of DSBs are a major part of the highly conserved ATM checkpoint pathway (Foster et al., 2006; Dimitrova and de Lange, 2009; Amiard et al., 2011). However, the telomeres of extant organisms use ATM-MRX/N (The yeast Xrs2 is replaced with NBS in all other organisms). The genetic characterization of telomere homeostasis in *Saccharomyces cerevisiae* led to the discovery of ATM-mediated anti-checkpoints. Similar schemes are likely to be present in most organisms, including *Drosophila* (Ciapponi et al., 2004; Gao et al., 2009).

In yeast, the ATM ortholog, Tel1 (Lustig and Petes, 1986; Greenwell et al., 1995), coupled with MRX, associate exclusively to short telomeres (Chang et al., 2007; Sabourin et al., 2007). These associations lead to telomerase activation. The counteracting inhibitory activities, Rif1 and Rif2, are recruited to longer telomeres. Rif1 acts to displace the Tel1 molecule, while Rif2 inhibits Tel1 binding to telomeric DNA (Martina et al., 2012). This feedback cycle continues whenever telomeres fall into a range that is sensed by an unknown mechanism to be too short or too long, creating a telomere size homeostasis. Such an equilibrium between mechanisms of telomere attrition and deletion and mechanisms of telomere elongation is present in both normal and oncogenic cells (Lustig, 2003; Pickett et al., 2011; Pickett and Reddel, 2012). Details of this model are far more complex (Sreesankar et al., 2012). For example, Rif1 and Tel1 operate by altering the timing of replication (Peace et al., 2014; Sridhar et al., 2014) and, very likely, TBP binding is regulated temporally within the context of the cell cycle.

#### The NHEJ Protein Ku Complex Obstructs the Formation of Telomere Fusions

The third conserved feature of telomeres is terminal capping. One of these complexes is the Ku70/Ku80 heterodimer (Polotnianka et al., 1998; Baumann and Cech, 2000). Ku, paradoxically, plays a vital role in non-homologous recombination of blunt-ended DNA damage. However, Ku can also act as an inhibitor of ligation at telomeres. Indeed, Ku70/Ku80 acts to prevent the deleterious ligation of two telomeres. Inhibiting the formation of dicentric chromosomes (Polotnianka et al., 1998; Williams and Lustig, 2003). Dicentric chromosomes undergo a series of breakage-fusion-breakage cycles, as observed in maize (McClintock, 1942). While higher plants tolerate this damage during mitosis, very few other organisms are resistant to this process. Dicentric chromosomes in most organisms fail in meiosis.

#### CST, the Telomeric RPA Complex?

The terminal CST capping complex mimics the structure of Replication Factor A (RPA). However, their activities are functionally distinct (Wellinger, 2009). CST, as RPA, acts at multiple genomic sites (Miyake et al., 2009). However, rather than acting as a telomeric cap, RPA stabilizes single-stranded DNA at the telomere and elsewhere (Price et al., 2010; Chen et al., 2012; Wang et al., 2012). Both RPA and CST form complex trimeric structures but only contain small patches of sequence homology. However, crystal structure analyses have shown that the RPA2 and STN1 subunits of RPA and CST, respectively, have very similar structures, as do RPA3 and TEN1 (Sun et al., 2009). The maintenance of protein structure is also responsible for interaction in the absence of extensive homology. Given the prevalence of both CST and RPA in all eukaryotes, ancestral RPA subunits may have formed paralogs that subsequently diverged in primary sequence, while maintaining the structure of the RPA and CST subunits. In reality, this is probably often the case, but is usually reflected in the primary sequence. Hence, these “structural” paralogs can be missed in the absence of extensive sequence homology.

#### Telomeric Repeat-Containing RNA (TERRA) and T-Loops: Conserved Nucleic Acids

Several nucleic acids play important structural roles at many telomeres. First, t-loop structures, the result of intrachromatid invasion of the telomeric terminus into more proximal sequences, remain stable and may hide the single strand from telomere addition. It may also act as either a structural block or part of the telomere replication process (de Lange, 2002; Luke-Glaser et al., 2012). Second, in most organisms, unique telomeric repeat-containing RNA (TERRA) transcripts are initiated within a subtelomeric element and proceeds in a 5′ to 3′ direction toward the terminus. Very little is known about the function of TERRA at the telomere. (Maicher et al., 2014). However, in exciting new research, G4 DNA acts synergistically with TERRA to form complex structures, some of which could extend or shorten the telomere (Xu, 2012). TERRA also appears to regulate the very short and elongated telomeres of the alternative pathway of telomere addition (Arora and Azzalin, 2015). TERRA may protect the telomere and regulate telomerase addition, as well as participate in non-telomeric functions.

#### The Conservation of G4 DNA *In Vivo*

G4 DNA consists of non-canonical Hoogsteen base paired structures present in the high G + T content of the telomere. The formation of these structures has been postulated to be a conserved element in the evolution of telomeres. The evidence for the presence of G4 DNA is its ability DNA to bind unique ligands and clear histones from promoter regions.

G4 DNA can form at both regular and irregular repeated telomere sequences (such as yeast) *in vitro*. There is strong evidence for the function of G4 DNA at the telomere *in vivo*. In general, G4 DNA has a protective function, albeit redundant with other overlapping functions. G4 DNA also has a high binding affinity for Mre11. For example, in the absence of the normal capping mechanisms, G4 DNA can block exonuclease function (Smith et al., 2011). Both findings are consistent with the view that G4 DNA served as an initial cap early in evolution (Garavis et al., 2013). In some contexts, G4 structures alone can have a deleterious effect. For example, in yeast, the coating of the single-strand overhang with RFA prevents the interference of G4 structures with lagging strand semi-conservative DNA synthesis (Audry et al., 2015). Cdc13 has also been implicated as a G4 TBP, given the simultaneous loss of a G4 DNA cap function only in *cdc13-1* cells (Smith et al., 2011).

Both positive and negative G4 functions at the telomere have been substantiated in the context of a vast number of other studies. Studies in the ciliate Oxytricha provide the best evidence for a positive function of G4 DNA *in vivo*. Under a complex set of interactions between the major two telomere proteins, TEBF alpha and TEBP beta, TEBP beta coupled with G4 DNA structures can facilitate telomere elongation (Oganesian et al., 2006). Indeed, the G4 structure may serve as a primer for telomerase. These studies recapitulate earlier *in vitro* findings (Fang and Cech, 1993b). Similarly, G4 DNA in humans acts as a positive regulator of telomere elongation (Moye et al., 2015).

As noted, the presence of G4 DNA is not restricted to the telomere, but has activity in other regions. These regions include chromatin enriched for rDNA and promoters of genes encoding both transcriptional regulators and telomeric proteins (Paeschke et al., 2005). Indeed, Sgs1 helicase is required for transcriptional activation, suggesting that unwinding of the G4 DNA is needed for activation (Hershman et al., 2008). Supporting this view, multiple experiments in yeast and humans have shown that both Sgs1 and Pif1 helicases bind to and unwind the G4 DNA conformation (Han et al., 2000; Budhathoki et al., 2015; Duan et al., 2015). G4 DNA binding proteins (G4BP) are also likely to be regulators of telomeres through their action at promoters. Hence, the telomere may be influenced either directly through G4BP binding or indirectly through the regulation of the transcription of a TBP. Telomeric imperfect repeats can also form G4 structures that are thermodynamically distinct (Lustig, 1992). What is not known is what type of Hoogsteen base paired structures forms *in vivo*.

### The Minimal Modular TBP

**FIGURE 2:**
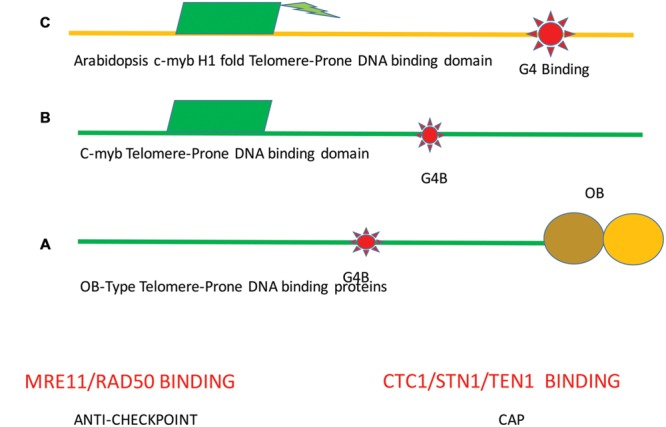
**Minimal function *cis*-acting elements at the telomere.** A minimal telomere consists of different combinations of modular domains. **(A)** MR, referring to the MRX and MRN (duplex binding) complexes and the CTC complex that binds to single-stranded are required telomeric DNA binding proteins that are common to the DNA of most telomeres. Both proteins will result in far greater stability. **(B)** In the second module, the DNA duplex binding is mediated by c-myb binding sequences. TBP will also bind to G4DNA structures. Of course combinations of these modules are possible and in both cases MR binding to duplex DNA and the Cst1/Stn1/Ten1 (CST) binding to single stranded DNA will increase stability. Green indicates c-myb. This model is similar to the one proposed by Linger and Price (2009). Each modular protein can mutate or recombine with other modules to give rise to the possibility of modular based mutagenesis. **(C)** In plants, a different module is found. A single essential structure is formed that contains the c-myb domain with histone H1 folds to bind duplex DNA. Also, TBP associates with G4DNA. Other proteins that are present in plants and bind to ends include the MR and CTC complexes as in animal and fungal species. Ultimately the MR structure provides equilibrium of telomere sizes that serve to protect the end from double-strand break processing enzymes, resulting in the anti-checkpoint.

Previous investigators have postulated the least number of modules for a common functional TBP (Linger and Price, 2009). These modules consist of at least a c-myb (dsDNA) and/or an OB (dsDNA) binding motif. In plants, a c-myb/histone H1 binding domain is a frequent telomere-binding element (Hwang and Cho, 2007). Hence, the combination of the DNA binding domains and G4 structures should be considered as an in *cis* telomere motif that has an essential role at the telomere. Many proteins that play widely different cellular roles can associate with one or more modules (**Figure 2**).

This modular structure helps to explain the finding that primary ciliate TBP (TEBF beta), the yeast Cst1 (Cdc13), and the human PPT1 TBP bind both to single-stranded DNA via OB-folds. Analogously, TEBF alpha shares homology with POT1 and binding to single-stranded DNA (Xin et al., 2007). G4 structures recruit MRX in yeast, thereby providing a source for homeostasis and a telomeric cap (Ghosal and Muniyappa, 2005). Whether this is a common phenomenon is not yet known.

### The Diverse and Variable TBP: The Role of Stress Response and High Selection Pressure in Diversity

Stress response at the level of the cell cycle may initiate selection over an evolutionary time scale. In the context of the cell cycle, cells carrying a non-functional TBP may lead to dysfunctional telomeres that respond through a cellular stress mechanism. Results from the Lundblad lab suggest that after telomere loss, but before significant telomere loss, pathways with differential dependencies on telomeric regulators produce differing pathways of senescence (Ballew and Lundblad, 2013). Moreover, microarray studies reveal a major reprogramming of global gene expression after the loss of telomerase (Nautiyal et al., 2002), We have also generated evidence that argues for two pathways that retard the rate of senescence *in vivo*: the DSB and replicative repair pathways. The attempts to repair continue even under senescent conditions. These pathways may also be required in wild type cells. These data argue for multiple senescence-specific telomere loss pathways (Gao et al., 2014).

The physiological states that have conferred known cellular stress responses include replication stress response, heat shock stress response, and the oxidative stress response pathway. The oxidative response, for example, induces pathways that prevent the damage created by free radicals to a multiplicity of substrates. One of the response factors is the Ogg1 DNA glycosylase that catalyzes the repair of base excision damage induced by oxidation (Lu and Liu, 2010). Interestingly, *ogg1* mutants confer elongated telomeres, raising a possibility of a link between oxidative stress and telomeres (Akerfelt et al., 2010; Lushchak, 2011). In bacteria, the SOS response to massive DNA damage includes the activation of *recA* that coats single-stranded DNA and allows DNA repair (Witkin, 1991). The *recA* response clearly shows that stress response are common in all phyla (Jin et al., 2015).

We propose a stress response for telomere dysfunction that acts over an evolutionary level time frame. The telomere dysfunction would lead to a more continuous period of enhanced recombination and mutagenesis. In this context, cellular stress would be maintained through multiple generations. Several investigators have provided evidence for an elevation in recombination and mutagenesis in response to telomere replicative DNA stress (Shor et al., 2013; Meena et al., 2015). There is also evidence for TERRA-mediated replicative stress (Lopez de Silanes et al., 2014). Specifically, TERRA might participate in DNA-RNA G4 structures at telomeres and, at Watson-Crick based paired R-loops, forming G-loops (Duquette et al., 2004) The possibility of a G4 R-loop that could impede replication has also been a topic of speculation (Xu and Komiyama, 2012).

The induction of recombination under telomere stress could give rise to additional duplication events. One member of this pair would encode the progenitor protein of a telomere-independent nuclear chromatin protein (such as Orc1) that is maintained under selection. The second copy would be free to diverge into a TBP from Orc1. Alternatively, duplicated DNA encoding two diverged TBPs may alter their telomeric roles. We also propose an elevated rate of mutagenesis allowing rapid sequence divergence. In some situations, only a few essential residues may be necessary to form a distinct protein function. Following multiple generations under stress, partially stable proteins can attain incremental changes in protein function.

What might be a signal for a stress response that initiates the rapid evolution of TBP? For a signal to be effective, cells must be acutely sensitive to multiple indicators of telomere function. These indicators must measure parameters including (a) the state of the leading and lagging strands of semi-conservative replication, (b) the activity of telomerase, (c) the non-nucleosomal telomeric chromatin structure, (d) telomere size changes, (e) the nucleosomal subtelomeric heterochromatic state, (f) telomeric G2 cohesion, and (g) non-disjunction. We believe that the unique integration of telomeres into many cellular processes that contribute to and are influenced by telomere function may increase the rate of TBP evolution. The degree of telomeric damage cannot be so severe that the defect induces a cell checkpoint pathway within a single cell cycle. Rather, subtler defects may induce a response that leads to the formation of paralogs and novel factors that can resolve the stress over evolution.

Different modules may also respond differentially to stress response or selective pressure. An intramolecular recombination event with a homolog may lead to exon shuﬄing among the TBP. An additional class of paralogs may have domains that are differentially influenced by mutations (see Sir3 discussion below).

In addition to paralog formation and high levels of mutagenesis, rapid alterations in proteins can result in simple substitutions of other known proteins as well as protein loss. The data that support the former viewpoint has arisen from close examination and experimentation of the primeval yeast whole-genome duplication (WGD; Hufton and Panopoulou, 2009). According to one Bayesian analysis of paralogs, WGD tends to be involved in generating paralogs of a similar function (Guan et al., 2007). However, a recent study has revealed that paralogs formed after yeast WGD undergo a wide range of divergence (Soria et al., 2014).

## Evidence for Elevated Paralog and Mutations in the Rapid Evolution of TBP-A Yeast Case Study

### Gene Duplication and Divergence of One Paralog

#### Orc1 Paralog Formation with Sir3 in Budding Yeast

**FIGURE 3:**
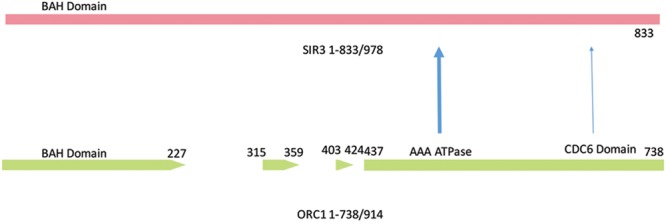
**Domain structure of Orc1 and Sir3.** The paralog, SIR3, shares the highly conserved Bromo adjacent homology (BAH) domain associated with transcriptional regulation, the AAA-type ATPase, and Cdc6 site. The sequence homology of ORC1 ends at amino acid 738 out of 914 residues. The Orc1 gaps are amino acids that differ between Orc1 and Sir3. The CTD of Sir3 (amino acids 820–978) is predicted to have a winged helical structure similar to Cdc6. The winged helix motif plays a role in DNA binding and protein/binding recognition, including the associations of histones H3 and H4. The numbered amino acid sequence refers to Orc1 amino acid number.

The yeast WGD serves as an outstanding model system for the study of the processes that lead to paralogs of differing function (Soria et al., 2014). An example (**Figure 3**) that has been examined in multiple fungal species (Capaldi and Berger, 2004) is the Origin of Replication Subunit 1 (Orc1). One of the paralogs of Orc1 in *S. cerevisiae* [and very closely related species (e.g., *S. byanus*)] is the Silencer Information Regulator 3 (Sir3; Liaw and Lustig, 2006).

Sir3 is a unique nuclear chromatin protein that functions in mating type and telomere silencing protein. In its mating-type silencing role, Sir3 maintain two of the three cassettes of mating-type information in a silent state, leaving only one of the cassettes in an expressed state (Lustig, 1998). Sir3 is essential for maintaining, but not establishing, the silencing of *HML* alpha and *HMR* a, present close to the left and right telomeres of chromosome III, respectively. Studies are conducted in the absence of mating type switching by using strains that lack the homothallic switching: gene, *HO*. In *ho* cells, incapable of mating type switching, only one mating type allele is expressed in haploid cells in the presence of the Sir3-dependent silent cassettes. The mating of *ho* haploids of different mating types produces diploids, permitting meiotic analyses. Meiosis is, of course, a significant selective force in evolution.

Sir3 is also essential for the silencing of ectopic telomere-adjacent genes associated with heterochromatic regions, a process termed telomere position effect (TPE; Gottschling et al., 1990). It is unlikely, however, that TPE plays a large role in cells lacking the ectopic silencing marker. Rather TPE is a quantitative read-out of the magnitude of heterochromatin formation in subtelomeric regions. In that regard, Sir3-dependent fold-back structures form at the subtelomeric/telomeric junction during maintenance of heterochromatin, a conclusion based on genetic and biochemical studies (Hecht et al., 1996) The fold-back structures resulted in homodimerization and heterodimerization of Sir3 and Sir4 in the telomeric regions and between telomeric and subtelomeric regions. At these sites, the heterochromatic proteins Sir3 and Sir4 interact with the C-terminal domain of the telomeric Rap1, and with N-termini of histones H3 and H4 (Kitada et al., 2012). Sir3 may also be important for the deletion of potential t-loops that may serve a sizing and protective functions (Bucholc et al., 2001)

Both the paralog Sir3 and the Sir4 protein associate with heterochromatic condensed chromatin and are necessary for maintenance, but not the establishment of silencing and heterochromatin. At higher concentrations, Sir3 has the unique property of spreading heterochromatin over an increasing distance from the telomere, a classic feature of eukaryotic heterochromatin (Buchberger et al., 2008)

The yeast Orc1 protein is a 914 amino acid (aa) protein with strong overall homology to other fungal Orc1 species. Orc1 contains the bromodomain adjacent homology (BAH) domain, an AAA ATP activity, and a Cdc6 winged helix domain (Wang et al., 1999; Capaldi and Berger, 2004). Orc1 has many of the features that are required to associate with the chromatin present during the initiation of DNA replication (Jiang et al., 2007; Prasanth et al., 2010; Thomae et al., 2011; **Figure 3**). Sir3 has 50% amino acids identity or similarity with these domains of Orc1. The most diverged portion of Sir3 primary sequence from Orc1 sequence is the 145aa C-terminal domain (CTD) present in Sir3. We have defined the CTD by the terminal sequences and the silencing activity displayed when the CTD is tethered to a specific chromosome (tethered silencing) and does not refer intrinsically to any structure (Liaw and Lustig, 2006; **Figure 3**).

**FIGURE 4:**
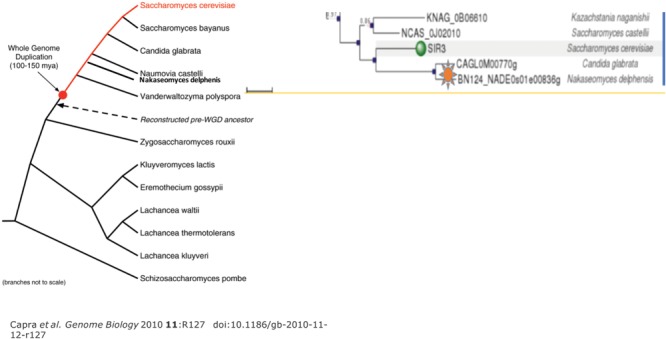
**The fungal phylogenetic tree shows the two pathogenic species.** On the left is shown the phylogenetic map for fungi showing the point of WGD for clarity. On the right is a tree rooted in similarity to *S. cerevisiae* Sir3 that is discussed in the text. Green indicates the *S. cerevisiae* Sir3 and *S. byanus*. Two lines below are depicted by the orange star are the *Candida glabrata* and *Nakaseomyces delphensis* strains. All strains are part of the ancestral WGD.

The CTD has been investigated by (a) a tethered silencing assay of the domain containing Sir3 in trans, (b) CTD crystallization, (c) CTD mutational analysis, and (d) a study of the CTD in context of the full length protein (Liaw and Lustig, 2006; Oppikofer et al., 2013). Two major conclusions can be drawn from these studies. First, the CTD contains a dimerization domain composed of a winged helix structure. Second, the CTD has a mutation of unknown function that is likely to be redundant within the full length Sir3. This structure is likely to be required for the assembly of histones and Sir gene products (Liaw and Lustig, 2006). In addition, Both Orc1 and Cdc6 maintain residual function in tethered silencing assays, suggesting a significant, but insufficient, role of the Orc1 and Cdc6 winged helix in silencing (Liaw and Lustig, 2006). Cdc6 can also physically associate with Orc1, but not with Sir3 (**Figure 4**).

**FIGURE 5:**
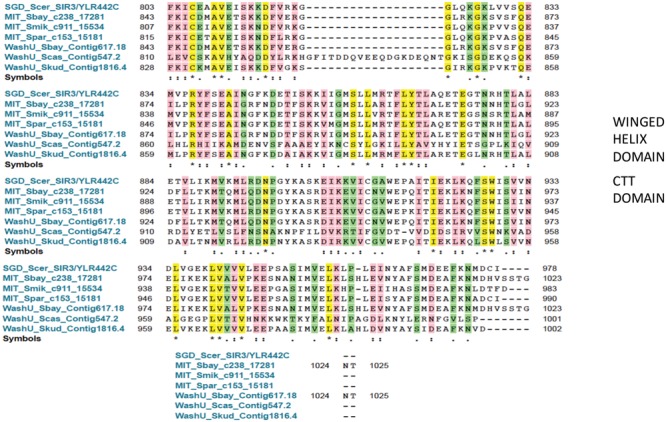
**Sequence Homology of CTD (CTT) in differing Saccharomyces species.** The lines (from top to bottom) display Sir3 from *Saccharomyces cerevisiae* sequence, *S. byanus, S. milkatae* a, *S. paradoxis*, an independent *S. byanus, S. castilli*, and *S. kudriavzevii*. Yellow refers to identity, pink to high similarity, and green to statistical similarity.

A close relative of *S. cerevisiae, S. byanus* can substitute for ScSir3 in a mating assay, despite its minimal CTD homology to ScSir3. We would predict that that domain of CTD also forms a winged helix domain, although this is uninvestigated. Such a rapid change in residues, however, may be due to a neutral effect of indels (mobile integrants) after the high levels of mutagenesis during the evolution of Sir3 (**Figure 5**).

#### Orc1/Sir3 Paralog Formation in Other Fungi

Our current studies show that a different form of Sir3 present in the Orc1 progenitor results in a pathogenic relative of *S. cerevisiae, Candida glabrata*. While ScSir3 behaves as a silencing protein (Liaw and Lustig, 2006), Cg Sir3 functions in a more elaborate silencing of many of the eicosapentaenoic acid (EPA) adherens. The adherens are under both positive and negative control for pathogenicity (Rosas-Hernandez et al., 2008; Halliwell et al., 2012).

**FIGURE 6:**
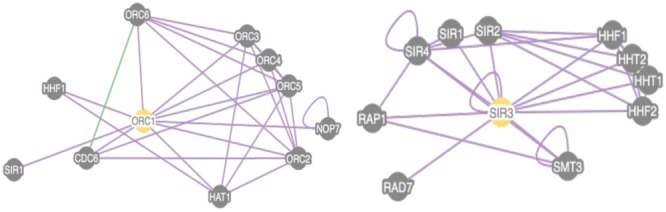
**Proteomic view of Association of Cdc6 with both Orc1 and Sir3 Protein-based associations are present between Orc1 and other yeast nuclear factors.** We conducted an SGD search for physical interactions between Orc1 or Sir3 and other cellular proteins using at least four experiments. Orc1 is capable of associating with Cdc6 while Sir3 is not. In no experiment was Cdc6/Sir3 binding observed. One genetic interaction between Orc3 and Orc6 is also shown in this figure.

**FIGURE 7:**
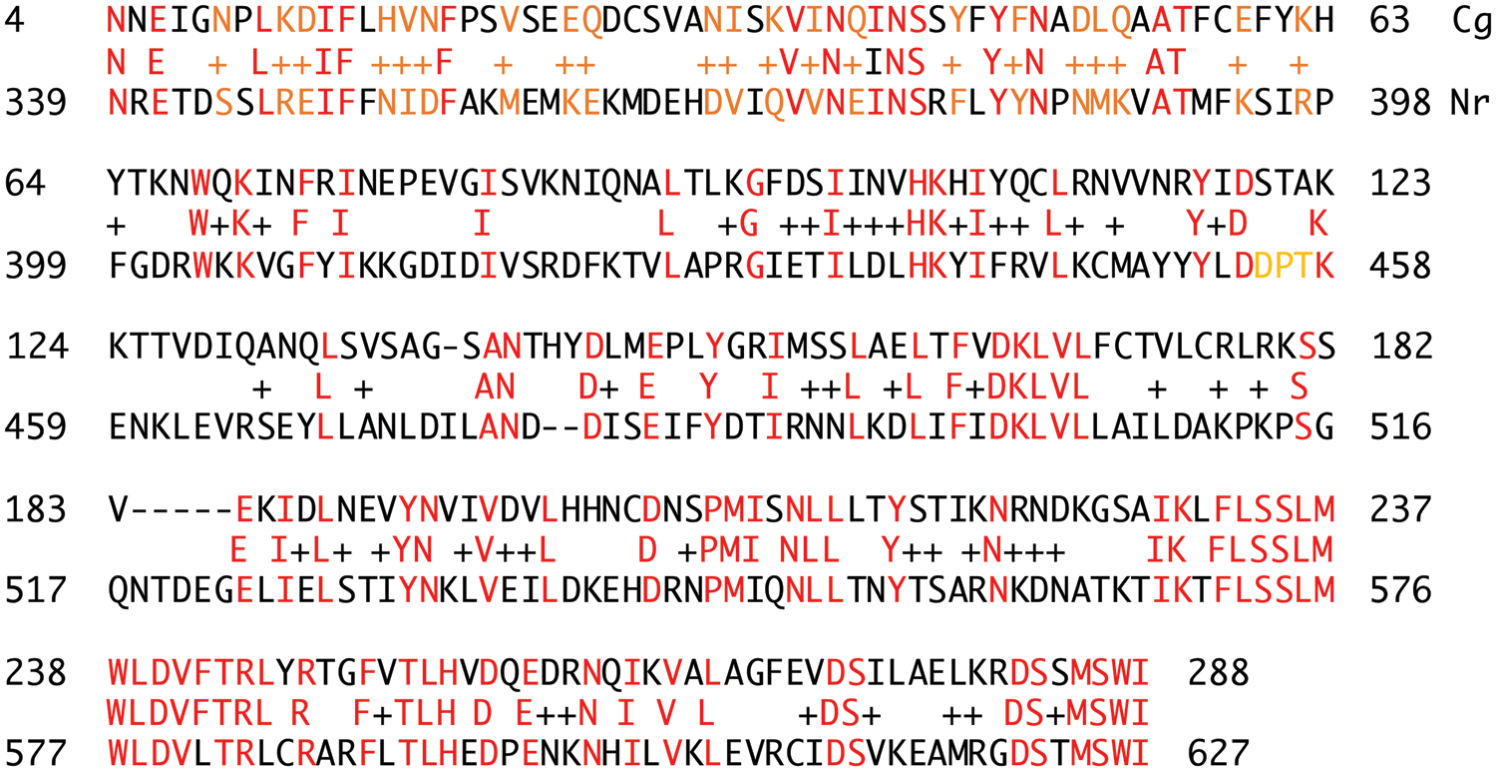
**High sequence similarity is present between *C. glabrata* (Cg) and *N. delphensis* (Nr).** The two different CTD sequences are shown in two close pathogenic species related to *Candida*. Red nucleotides shown identity and orange residues show similarities.

Interestingly, the adheren silencer is very close to the telomere, implicating functional involvement (Liaw and Lustig, 2006). Pathogenicity is also dependent upon other telomeric proteins, including Ku and Rif1. Each telomere of *C. glabrata* behaves differently in the context of silencing. The cgSir3 CTD is divergent from ScOrc1 or ScSir3 (**Figures 6** and **7**). We analyzed the Sir3 phylogenetic tree using Phylome DB (www.phylomedb.org) (Huerta-Cepas et al., 2008; Huerta-Cepas et al., 2014; **Figure 7**). Curiously, *C. glabrata* and the closely related pathogen *Nakaseomyces delphensis* have very similar CTD domains, but both are highly diverged from *S. cerevisiae* Sir3 CTD to the level of insignificance (Lustig, unpublished data). We therefore have operationally termed this region the CTD2 region. The altered CTD2 function undoubtedly responds to a different set of selective pressures, the expression of EPA adherens that are necessary for pathogenicity (Ielasi et al., 2012). The *C. glabrata* obligate haploid also has three mating type cassettes, reminiscent of ScSir3, but not involved in mating type identity. Nonetheless, one of these near the telomere is also is under the control of Sir3 at a transcriptional level, remnants of a system that may be in the process of evolving into a new function (Yanez-Carrillo et al., 2014). Additional selection pressures, yet to be deduced, may be present to influence CgSir3. The functional residues of CTD2 have not been studied (**Figure 7**). Study of this region also suggests that CgSir3 in *S. cerevisiae* and *S. glabrata* have an ancient common ancestor.

The CTD, in this case, would not be expected to be highly sensitive to mutagenesis, since the function of active sites can be perturbed by only a few single mutations. However, CTD2 may be similar to CTD1 in providing a mutational buffer against functional change. Both types of Sir3 diverged from Orc1 after whole genome duplication. Alternatively, although remote, Orc 1 may act independently but at high levels in paralog formation. In either case, the two forms of Sir3 may have diverged rapidly to produce the extant unique proteins (Fabre et al., 2005). Heterochromatin proteins in other organisms (Sugiyama et al., 2005), such as HP1 of *S. pombe*, share homology and function between centromere and telomere heterochromatin but have no evolutionary relationship to Sir3.

Thus, the Orc1/Sir3 system appears to be capable of two functional changes via the Sir3 CTD domain. Although a micro-evolutionary case, the paralogs are well suited examples of proteins with differing function. We propose that the elevation of paralog formation and mutagenesis at an evolutionary scale can promote rapid deviations in the related strains. Indeed, the divergence in CTD1 and CTD2 supports such an enhanced level of mutagenesis. Finally, we propose that this rate of adaptation is likely due to a yeast stress response that elevates recombination and mutagenesis.

### The Separation of Two Telomeric Functions by Gene Duplication: Est1/Ebs1

Sir3 is not the only example of a paralog that can lead to altered activity after WGD. Est1, a part of the telomerase holoenzyme, has a paralog, Ebs1 (Zhou et al., 2000; Luke et al., 2007). Ebs1 is a component of the non-sense-mediated mRNA decay pathways. Indeed, non-sense-mediated mRNA decay reduces telomere size (Lew et al., 1998). Ebs1 shares only 27% homology with Est1 throughout the protein, so that the conserved domain involved in size control remains ambiguous. Ebs1 is also present in a single Est1/Ebs1 protein in the more distant pre-WGD *Kluyveromyces lactis* (Hsu et al., 2012). This fusion protein is likely to be closer to the common ancestral precursor protein. The precursor must have produced paralogs during or after WGD, diverging into separate ScEbs2 and ScEst1.

### What Happened to RAP1? The Argument in Favor of Hypomorphs!

Most Rap1 molecules share the Rap1 C-terminal (RCT) domain (Chen et al., 2011). Rap1, in yeast, serves as the major functional yeast TBP that also is a DNA binding protein and a transcriptional activator of glycolytic and ribosomal protein genes (Shore, 1994; Park et al., 2002). A great deal of evidence has amassed for the function of mammalian RAP1 through multiple assays (Li and de Lange, 2003; O’Connor et al., 2004; Bae and Baumann, 2007; Bombarde et al., 2010; Chen et al., 2011; Arat and Griffith, 2012) and is the most conserved protein at the telomere (Yang et al., 2011; Martinez et al., 2013; Yeung et al., 2013). However, recent data revealed the unexpected result that loss of RAP1 in both mice and humans had no functional impacts at telomeres, but only in transcription (Martinez et al., 2010; Kabir et al., 2014). This could be the result of a requirement for the role in promoter activation in a limited number of transcripts (Bae and Baumann, 2007; Bombarde et al., 2010; Arat and Griffith, 2012) or the presence of a redundant telomere Rap1-like protein. Rap1may be present then at human telomeres as an artifact of the conserved heterodimer, TRF2/Rap1, at some promoters (Kabir et al., 2014).

How could RAP1 make such an evolutionary leap? Is this really due to a lack of function at telomeres? There are two other possible considerations. First, the RCT domain that is similar to the fission yeas *S. pombe* associates with the TRF2-like protein, Taz1, where deletion mutants have shown a high level of telomere involvement (Park et al., 2002). It seems unlikely that the lack of nucleosomes in *S. pombe* telomeric chromatin and its presence in human telomeres governs this loss of Rap1 activity, Rap1 binding occurs via Taz1 and can function transcriptionally on nucleosomal DNA in mice or human cells (Wright et al., 1992; Park et al., 2002; Tomaska et al., 2004b; Galati et al., 2012).

We propose a number of solutions to this odd situation. The first, functional redundancy, is unattractive in its simplest form, since its presence would mask the phenotypes of rap1 mutants. Rather, we make a second proposal, albeit speculative, based on the inability to explain conservation in the absence of selection. Similarly, the transcriptional function in human cells do not appear extensive enough to induce such a strong selection. We therefore suggest differences intrinsic to hypomorphic and null alleles. In the presence of a horrendous telomeric damage event, viable cells could produce a “defect response system,” not unlike many of the responses to serious cellular defects. A previous observation noted that a loss of RAP1 led to an increase in recombination (Sfeir et al., 2010), consistent with this idea. As noted, in the yeast *S. cerevisiae*, there is some evidence for rapid effects on recombination and mutagenesis in the face of telomere disaster (Shor et al., 2013; Meena et al., 2015). Recombinational induction has also been observed rapidly in yeast without the expected DNA damage response pathway (Lustig, unpublished data), consistent with effect found in human cells. We would like to propose that there is a telomere response system that is distinct from the DNA damage response pathway that can sense (through an unknown signal) an alteration in essential chromatin structure. A null allele might simply place too much stress on the cell, promoting the induction of specific proteins, one of which may have some functions of RAP1. Possibly, more information would be gained by the use of hypomorphic mutations that retains partial Rap1 function that may not be susceptible to this putative response. Under these hypothetical, conditions, the telomere damage may be below the sensitivity of detection, circumventing the effect of the response system. Under non-null conditions, the true effects of Rap1may be better determined, one way or another. This issue may be raised for a number of observations that seem to be signaling effects, rather than the original transient effect of the mutation

## Complex Telomeres: Speculation on the Flexible Dynamics of Shelterin

We normally think of shelterin an ordered set of proteins that are invariant in humans (de Lange, 2005). Shelterin is an outstanding model system to discuss the numerous ways of attaining a broader level of control. The conservation of shelterin function is likely to be a consequence of the interaction between the functional subunits (de Lange, 2005) that contain common motifs such as c-myb, OB, and G4 modules. Also, it is likely to involve the formation of only a subset of protein/protein junctions that are sterically and thermodynamically permissible. In addition, a subgroup of chromatin-associated proteins, TRF1, TRF2, and POT1 has probably evolved through a paralog-related process. So the overall constraints of variable TBPs include geometry, protein/protein interfaces, and the presence of proteins having truly unique functions. This set of constraints will vary through evolution in species having a multi-subunit shelterin-like structures. The nature and frequency of the multi-subunit protein interfaces would select for only steric and thermodynamic limitations, based on protein folding structures that fit the geometric and functional needs of the telomere.

When honing in on vertebrates (or mammals), it is clear that TRF1 is the ancestral protein to vertebrate TRF1 and TRF2 paralogs (Horvath, 2008). Similarly, TRF2, a paralog of TRF1, has become substantially specialized. TRF2 plays multiple roles in telomere maintenance and dynamics that are due to the unique chromatin structure (Broccoli et al., 1997). However, the TRF1-nucleated class may have been derived by a TRF1 ortholog precursor to the major telomere proteins present throughout vertebrates (Horvath, 2008). Therefore, previous studies may not solve the telomere function in all complex vertebrates (except in mammals), but demonstrate one of many possible solutions that exist in extant organisms.

Paralog functions do play a role in some shelterin complex telomeres such as in the formation of Pot1a and Pot1b in rodents (Hockemeyer et al., 2006), but also in other organisms that have simpler telomeres, such as *Arabidopsis*, green algae, and the ciliate *Tetrahymena*. Pot1 forms homologs Pot1a and Pot1b in several species that are distant evolutionary, such as *Tetrahymena* (Jacob et al., 2007; Shakirov et al., 2009). The maintenance of the POT1 class of proteins is critical for shelterin function. POT1 plays a predominant role in the accessibility to and modulation of telomerase. Tankyrase, the protein that is responsible for the loading of TRF1 in vertebrates, also plays a role in plants. Importantly, this is a class of proteins with similar structure, but differing function, another possible outcome of paralog formation that both play a role at the telomere (Cook et al., 2002). In plants, tankyrases do not act as a TRF1 loading factors. That is not surprising given the evidence that TRF proteins are not functional in *Arabidopsis* (Boltz et al., 2014; Fulcher and Riha, 2015). A resolution of whether the tankyrases in plants are true paralogs and the nature of their specific function at telomeres will require future investigation. Telomerase holoenzyme also undergoes species-dependent paralog formation, particularly in Est1 and Pot1 (e.g., Est1a, b, c Pot1a, Pot1b). Est1a complements senescence in yeast and performs the telomerase function. The function of Est1b and Est1c are unclear (Sealey et al., 2011). Paralogs of Est1 are exclusively observed in humans. As expected, the conserved TBP components discussed in section “The Conserved Elements of TBP” are also present at human telomeres in addition to shelterin. This model coupling paralog formation and interface compatibility in the presence of a minimal number of conserved proteins is a proposal that tries to explain the rapid evolution of TBPs. Other ideas involving the cooperativity of processes are in no way mutually exclusive from our considerations.

Hence, the plethora of proteins present in a given cell type is likely to overcome a major thermodynamic barrier to the formation of shelterin. The formation of shelterin-like complexes may be the consequence of a trial and error process that may require sub-complexes. The shelterin complexes that are present in more complex organisms are under, as yet, uncharacterized selection pressures.

## A Model for the Rapid Evolution of Telomere Binding Proteins

We propose five central principles that serve as the foundation for the rapid evolution of telomere-binding proteins. First, paralog formation seems to be a primary driving force in rapid evolution rather than ortholog formation. Second, telomere-binding proteins consist of a limited number of conserved motifs such as c-myb, OB, and G4 domains, which can initiate a minimal level of protection. Third, stress response at the evolutionary level may occur as the result of telomere dysfunction that increases the rate of recombination and mutagenesis. Fourth, the major limiting function in complex shelterins is the number of protein/protein interfaces needed to form a multi-subunit complex-as least at the structural level. Specific required functions may be under additional selection pressure. Fifth, some complexes provide novel functions (e.g., Pot2 access to telomerase) and the transducing of signals over a large portion of the telomere that may have effects that are greater than the sum of individual protein species. These five principles serve as the basis of any attempt to create a coherent evolutionary model.

We believe that the vastly different organismal requirements may alter selection patterns. For example, the abundance of telomeres, the cell cycle control of replication, the coordination of telomere and semi-conservative replication may have profound effects on the nature of telomere change (Horvath, 2008).

**FIGURE 8:**
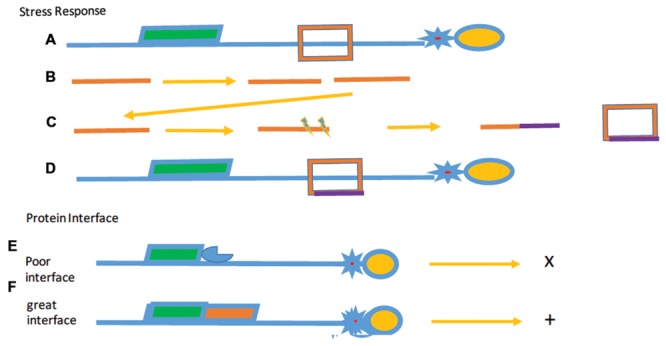
**Selected stress responses and surface interactions.** *Selective Stress* **(A–D)** shows the proposed effect of stress responses over evolutionary time. **(A)** The initial modular telomere with a TBP (red). **(B)** Duplication of DNA (orange) encoding the protein. **(C)** Separation and mutagenesis (purple) of the two DNAs and the altered protein (orange and purple box. **(D)** The final telomere, bound by the mutated protein. Protein Legend: c-myb containing protein: green, G4 DNA binding protein (star), and OB-fold protein (yellow). *Protein interfaces*. In example **(E)**, protein (blue) associated with the c-myb protein with an unfavorable surface interaction shown by the x. **(F)** Protein interfaces that interact favorably with a second protein (red) to form a stable structure as indicated by the +. A simplified minimal modular telomere is shown just for reference.

We propose, therefore, that the phenomenon of “rapid evolution” is the consequence of the high level of paralogs, producing distinct functional proteins through the induction of telomere stress response. While telomere evolution is clearly not the only case in which paralogs may evolve to form other functions, alterations in TBPs must be driven by the need for rapid response to physiological change (**Figure 8**).

A large number of experimental studies serve as the basis of these models. A complete solution to the patterns observed will require a greater knowledge of telomere protein/protein interactions and telomere protein domain structure. This level of understanding requires a collaborative effort to characterize more organisms for genetic analysis.

## Author Contributions

AJL is responsible for the content contained in the manuscript.

## Conflict of Interest Statement

The author declares that the research was conducted in the absence of any commercial or financial relationships that could be construed as a potential conflict of interest.
